# Supplementary figures and images for: Planarian stem cells sense the identity of the missing pharynx to launch its targeted regeneration
Source: eLife. 2021 Jun 22;10:e68830. doi: 10.7554/eLife.68830 (PMC8219383; doi:10.7554/eLife.68830)

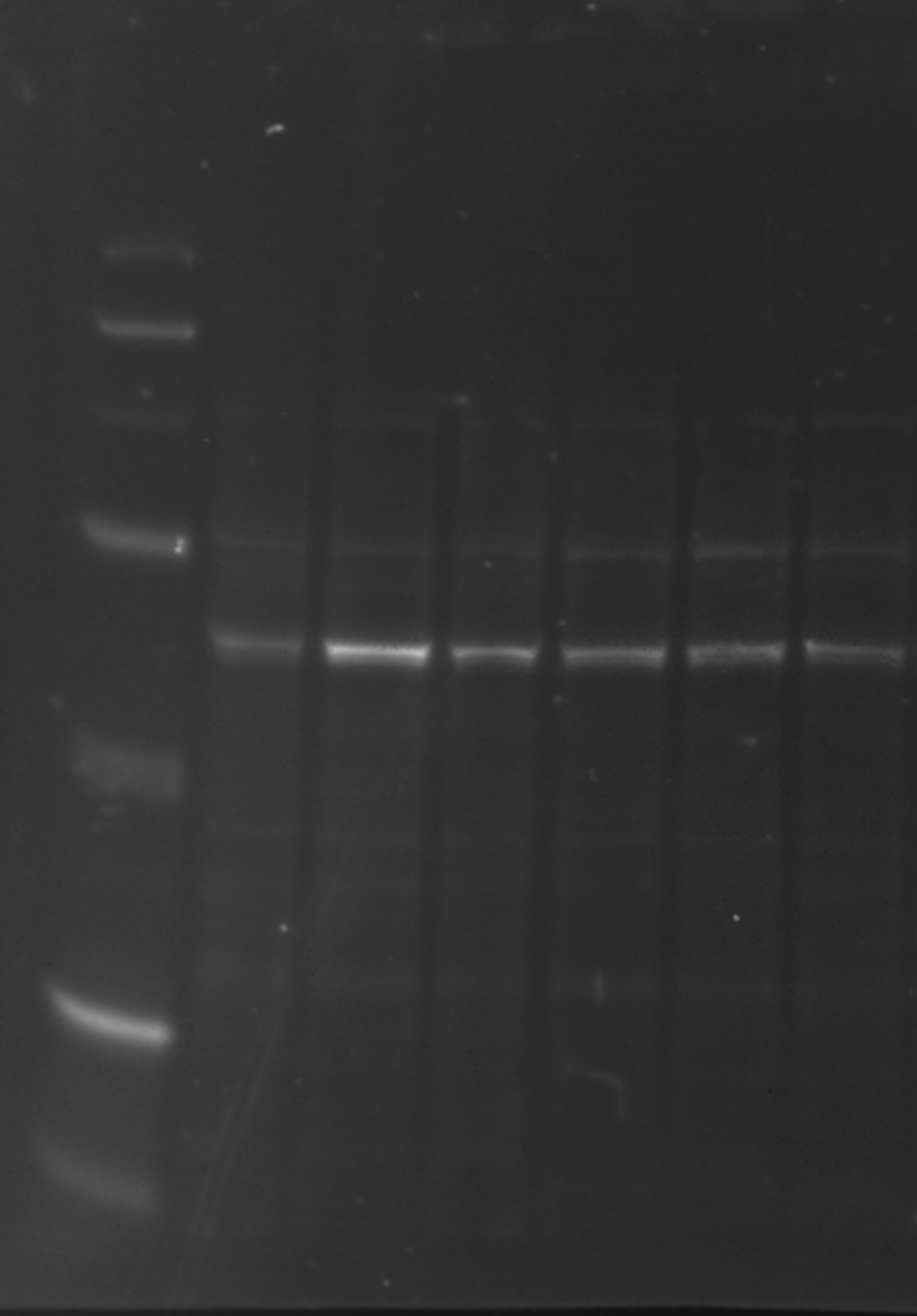

Supplement: Figure 5—source data 1. [file elife-68830-fig5-data1.zip › Figure 5-source data 1/Figure 5A raw (pERK).tif]

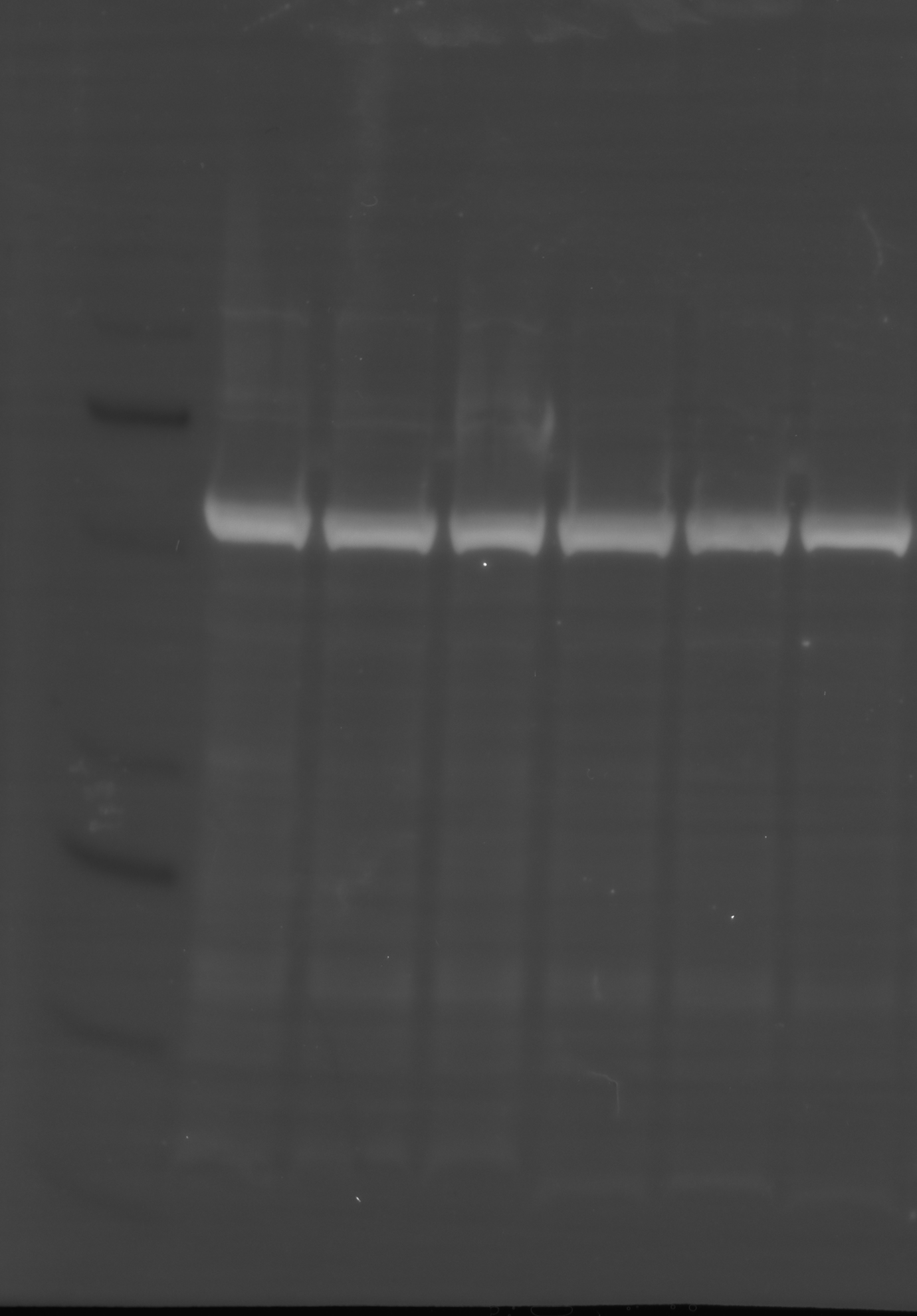

Supplement: Figure 5—source data 1. [file elife-68830-fig5-data1.zip › Figure 5-source data 1/Figure 5A raw (tubulin).tif]

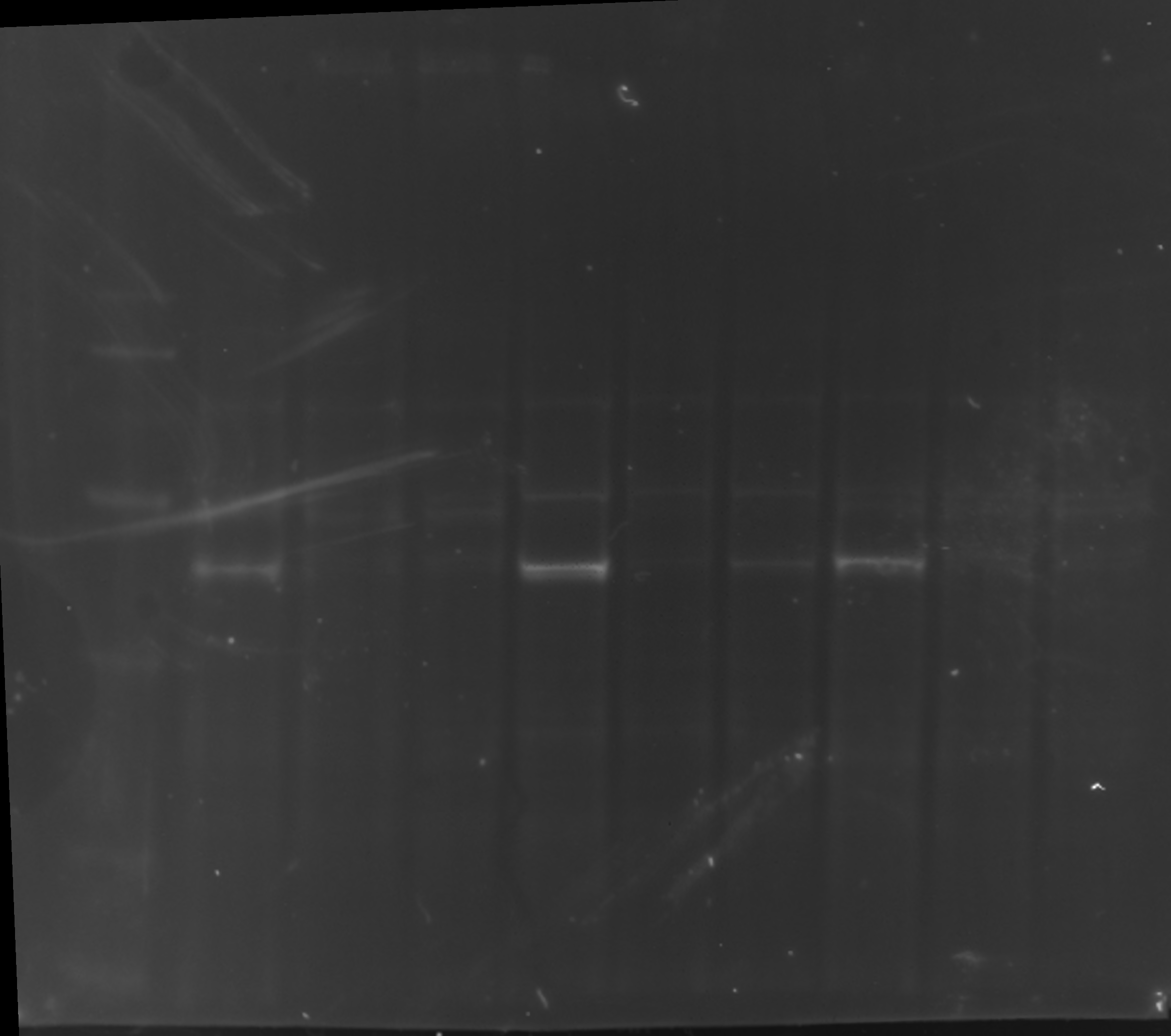

Supplement: Figure 5—figure supplement 1—source data 2. [file elife-68830-fig5-figsupp1-data2.zip › Figure 5-Supp 1-source data 2/Figure 5_S1E raw (pERK).tif]

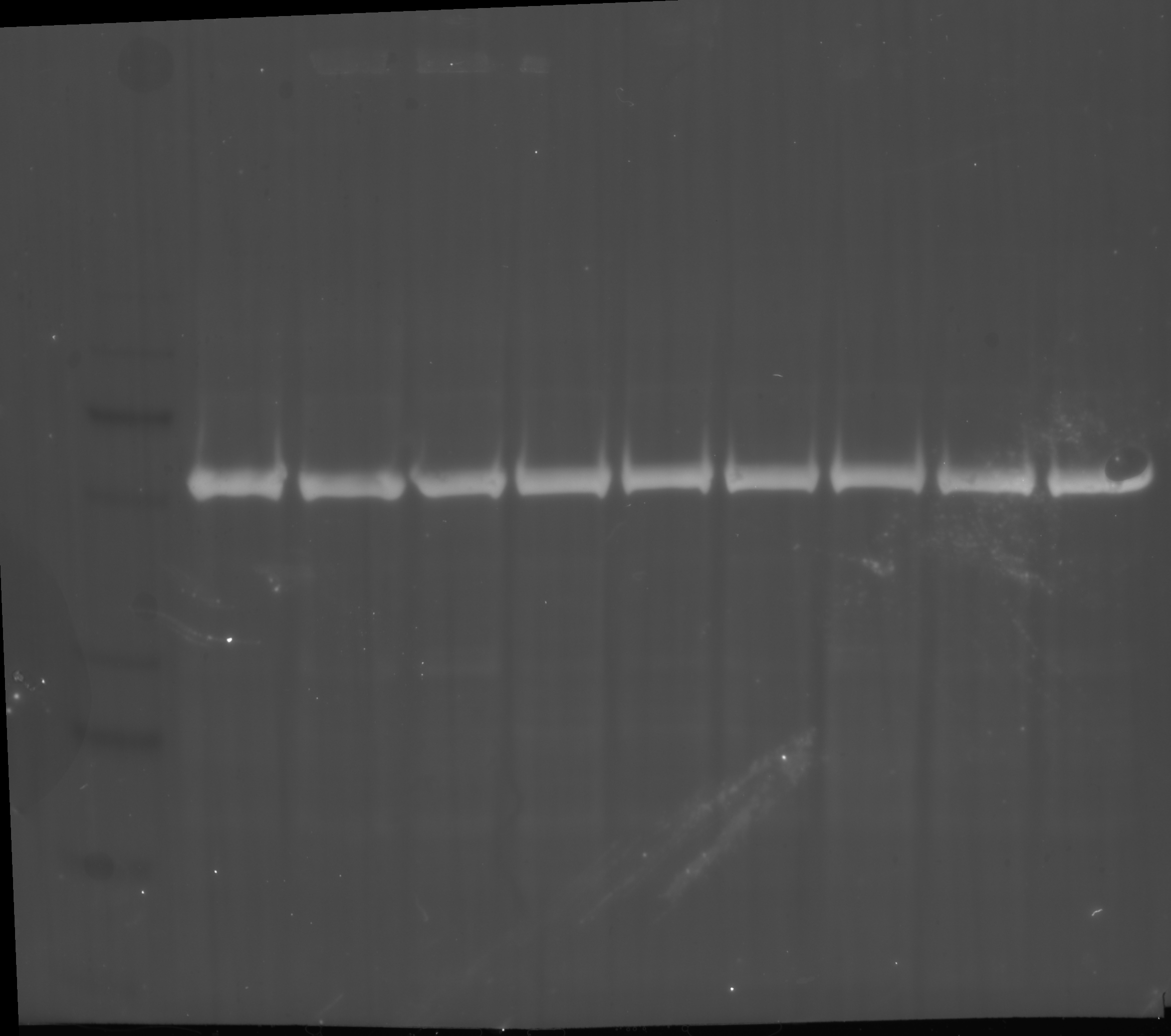

Supplement: Figure 5—figure supplement 1—source data 2. [file elife-68830-fig5-figsupp1-data2.zip › Figure 5-Supp 1-source data 2/Figure 5_S1E raw (tubulin).tif]
